# Comparative analysis of the complete sequence of the plastid genome of *Parthenium argentatum *and identification of DNA barcodes to differentiate *Parthenium *species and lines

**DOI:** 10.1186/1471-2229-9-131

**Published:** 2009-11-17

**Authors:** Shashi Kumar, Frederick M Hahn, Colleen M McMahan, Katrina Cornish, Maureen C Whalen

**Affiliations:** 1 Crop Improvement and Utilization Research Unit, Western Regional Research Center, ARS, USDA, 800 Buchanan Street, Albany CA 94710, USA; 2 Yulex Corporation, 37860 W Smith-Enke Road, Maricopa, AZ 85238-3010, USA

## Abstract

**Background:**

*Parthenium argentatum *(guayule) is an industrial crop that produces latex, which was recently commercialized as a source of latex rubber safe for people with Type I latex allergy. The complete plastid genome of *P. argentatum *was sequenced. The sequence provides important information useful for genetic engineering strategies. Comparison to the sequences of plastid genomes from three other members of the Asteraceae, *Lactuca sativa, Guitozia abyssinica *and *Helianthus annuus *revealed details of the evolution of the four genomes. Chloroplast-specific DNA barcodes were developed for identification of *Parthenium *species and lines.

**Results:**

The complete plastid genome of *P. argentatum *is 152,803 bp. Based on the overall comparison of individual protein coding genes with those in *L. sativa, G. abyssinica *and *H. annuus*, we demonstrate that the *P. argentatum *chloroplast genome sequence is most closely related to that of *H. annuus*. Similar to chloroplast genomes in *G. abyssinica, L. sativa *and *H. annuus*, the plastid genome of *P. argentatum *has a large 23 kb inversion with a smaller 3.4 kb inversion, within the large inversion. Using the *mat*K and *psb*A-*trn*H spacer chloroplast DNA barcodes, three of the four *Parthenium *species tested, *P. tomentosum*, *P. hysterophorus *and P. *schottii*, can be differentiated from *P. argentatum*. In addition, we identified lines within *P. argentatum*.

**Conclusion:**

The genome sequence of the *P. argentatum *chloroplast will enrich the sequence resources of plastid genomes in commercial crops. The availability of the complete plastid genome sequence may facilitate transformation efficiency by using the precise sequence of endogenous flanking sequences and regulatory elements in chloroplast transformation vectors. The DNA barcoding study forms the foundation for genetic identification of commercially significant lines of *P*. *argentatum *that are important for producing latex.

## Background

*Parthenium argentatum *Gray, commonly known as guayule, is a shrub in the Asteraceae that is native to the southwestern United States and northern Mexico. *Parthenium argentatum *produces high quality rubber in bark tissue, which is under development for biomedical uses. The U.S. Food and Drug Administration recently approved the first medical device made from *P. argentatum *natural rubber. Products made from *P. argentatum *latex are designed for people who have Type I latex allergies, induced by natural rubber proteins from *Hevea brasiliensis*. In addition to biomedical products, natural rubber is essential and irreplaceable in many industrial and consumer applications, and the price is rising under heavy demand, making natural rubber increasingly more precious. As an industrial crop that grows in temperate climates, *P. argentatum *represents a viable alternative source of high quality natural rubber.

One strategy for improving crops, such as the rubber-producing *P. argentatum*, is through chloroplast engineering [1-3]. Transformation of chloroplasts allows high-level production of foreign proteins because of the high number of chloroplasts per plant cell. As homologous recombination is the means by which foreign DNA is incorporated into the chloroplast genome, transformation is precise and predictable. Moreover, it has been shown that up to four genes can be inserted at once [4], enhancing the efficiency of metabolic engineering. From production of edible vaccines to bioplastics, transplastomic plants have been shown to provide a useful route to manipulate crops for industrial purposes [5].

Importantly from the point of view of minimizing environmental impact, expressing foreign proteins in the chloroplast results in transgene containment [6,7]. It is thought that in the vast majority of plant species, chloroplasts are not transmitted by pollen, and so in these species, chloroplastidic transgenes would not be spread in that manner. Although, it is becoming clear that each case must be thoroughly verified [8,9]. In the case of *P. argentatum*, transgene containment is important because it is currently cultivated as an industrial crop in its native region in the southwestern United States.

Construction of vectors for chloroplast transformation requires some knowledge of the chloroplast genome sequence to identify insertion sites. To date, just short of one hundred plastid genomes from angiosperms have been completely sequenced. The sequences are highly conserved [10]. Interestingly however, the order of genes in some groups, including the Asteraceae, Fabaceae and Poaceae, may be reversed by large inversions [11-13]. In the Asteraceae, the family of interest in this study, there is a second small inversion (~3 kb) nested within the larger inversion (~23 kb) [14]. The two inversions are always found together, implying that they occurred close in evolutionary time.

Chloroplast sequences are useful for identification of species, using a particular sequence as a DNA tag or barcode [15]. An ideal DNA barcode for general purposes would 1) have enough diversity to allow discrimination among species, but not so much that would prevent grouping of members of a species, 2) work in wide variety of taxa, and 3) provide the basis for reliable amplifications and sequences [16]. In plants, unlike in animals, the mitochondrial genome evolves too slowly to provide useful DNA barcode sequences. Although also possessing a relatively slow rate of evolution, several chloroplast sequences have been identified as fulfilling the criteria listed above [17-19]. Depending on the desired level of discrimination, the consensus conclusion appears to be that the low mutation rate in the chloroplast genome may require more than one barcode locus to be probed [18,20,21].

At present, classical breeding is being used to improve *P. argentatum *as a commercial source of natural rubber. Breeding efforts would be enhanced by informative chloroplast DNA barcodes. Because a very small amount of tissue is required for barcode analysis, purity of breeding lines can be determined at an early stage of seedling growth. In addition, barcodes would allow breeders and seed producers to discover seed lot contamination before advancing breeding lines for latex production. Having the ability to removing contaminating lines, especially when they represent lower rubber lines, would improve the efficacy of breeding efforts.

The focus of our research program is improvement of *P. argentatum *to enhance its commercial viability. We have chosen two approaches, biotechnology through chloroplast metabolic engineering and marker-assisted breeding. The *P. argentatum *chloroplast genome sequence that we report herein, supports our efforts in both approaches. In this article, we report the complete sequence of the chloroplast genome of *P. argentatum *and describe the development of DNA barcodes. The complete sequence of the *P. argentatum *chloroplast genome has enabled us to construct chloroplast transformation vectors based on the exact sequence of the large inverted regions, and to identify novel insertion sites in non-essential, non-coding regions. Barcode analysis with the *mat*K gene and *psb*A-*trn*H spacer sequence allowed us to discriminate three of four *Parthenium *species from each other and from *P. argentatum*, and a subset of the *P. argentatum *lines from each other. These barcodes will be used in our breeding program.

## Results

### Genome size and gene content, order and organization

**Figure 1:**
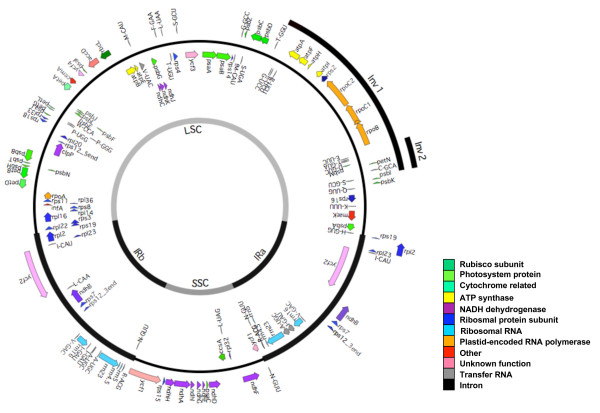
**Representative map of the chloroplast genome of *Parthenium argentatum *(Genbank Accession **GU120098**)**. IR, inverted repeat; LSC, large single copy region; SSC, small single copy region; Inv1, inverted sequence 1; Inv2, inverted sequence 2. Gene names and positions are listed in Additional file 1.

The complete nucleotide sequence of the chloroplast genome of *Parthenium argentatum *is represented in a circular map (Figure 1; Genbank Accession GU120098). It is 152,803 bp in size and includes a duplicated region of inverted repeats (IR) of 24,424 bp. The IR are separated by small single copy (SSC) and large single copy (LSC) regions of 19,390 bp and 84,565 bp, respectively. The total G+C content of the whole chloroplast genome is 37.6%. The gene content and arrangement were observed to be similar to those in *Lactuca sativa *and *Helianthus annuus *[22], and *Guitozia abyssinica *(NC_010601), including one large (Inv1) and one small inversion (Inv2) in the LSC region. There are 85 genes coding for proteins (Additional file 1), including six that are duplicated in the IR regions. There are four rRNA genes that are also duplicated in the IR regions. In total there are 43 tRNA genes, seven of which are duplicated in the IR, one in the SSC, with the remaining 28 scattered in the LSC region.

**Figure 2:**
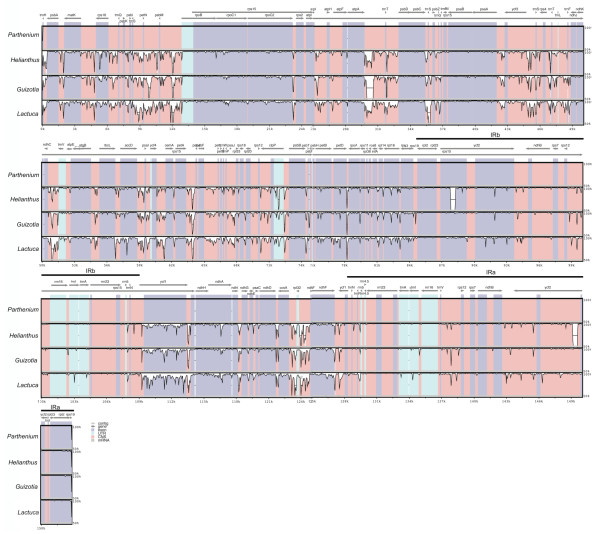
**Chloroplast genomes of *Parthenium argentatum*, *Helianthus annuus*, *Guizotia abyssinica *and *Lactuca sativa *compared with mVISTA**. A cut-off of 70% identity was used for the plot and the Y-scale represents the percent identity ranging from 50 to 100%. Blue represents exons, green-blue represents untranslated regions, and pink represents conserved non-coding sequences (CNS). Horizontal black lines indicate the position of Inv1, Inv2, IRa and IRb; SSC is flanked by IRa and IRb; grey arrows the direction of transcription.

The size of the *P. argentatum *chloroplast sequence is larger than those of the three other Asteraceae chloroplast genomes (Table 1). It is close to the same size as the *L. sativa *genome, and 1.04 kb and 1.7 kb larger than the *G. abyssinica *and *H. annuus *genome, respectively, with the length differences primarily found in the LSC and SSC domains. The sequence differences between *P. argentatum *and each of the other three chloroplast genomes are concentrated in the noncoding regions of Inv2, and the SSC and LSC regions (Figure 2). The IR regions in *P. argentatum *are shorter than those of the three other species by 210-610 bp (Table 1, Figure 2).

**Table 1:** Size comparison of *Parthenium argentatum *chloroplast genomic regions with those in other members of Asteraceae.

	Length (bp)			
	
Plant species	Total genome	LSC^a^	SSC	IR
*Helianthus annuus*	151104	83530	18308	24633
*Guizotia abyssinica*	151762	83636	18228	24950
*Lactuca sativa*	152772	84105	18599	25034
***Parthenium argentatum***	152803	84335	19390	24424

^a^Regions in chloroplast genome; LSC, Large Single Copy; SSC, Small Single Copy; IR, Inverted Repeats.

Based on sequence comparison of the chloroplast genome of *P. argentatum *with *H. annuus *and *L. sativa*, two inversions of 22,890 bp and 3,364 bp were observed in *P. argentatum*, similar to those described by Kim et al. [14] and Timme et al. [22]. In *P. argentatum*, one end point of the 23 kb inversion was located between the *trn*S-GCU and *trn*G-UCC genes. The other end point is located between the *trn*E-UUC and *trn*T-GGU genes. The second 3.4 kb inversion was observed within the 23 kb inversion, which shares one end point just upstream of the *trn*E-UUC gene with the large inversion. The other end point of the 3.4 kb inversion is located between the *trn*C-GCA and *rpo*B genes (Figure 1).

### Variation in chloroplast coding sequences of Asteraceae family members

Variation between coding sequences of *P. argentatum *and *H. annuus, G. abyssinica *or *L. sativa *was analyzed by comparing each individual gene (Additional file 1) as well as the overall sequences (Figure 2). In general, *P. argentatum *coding sequences are more similar to those in *G. abyssinica *(98.5% identical on average) and *H. annuus *(98.4%), than in *L. sativa *(97.2%). The greater average identity in *G. abyssinica *than in *H. annuus *is in large part due to deletions in the two copies of *the ycf*2 loci in *H. annuus*, otherwise, *H. annuus *is more similar overall than *G. abyssinica*. Fourteen genes in *H. annuus *and *G. abyssinica *were 100% identical to those in *P. argentatum*, compared to only four genes in *L. sativa *(Additional file 1). The most-divergent coding regions in the three genomes were *ycf*1, *acc*D, *clp*P, *rps*16, and *ndh*A (Figure 2).

### DNA barcode analysis of Parthenium

To differentiate *Parthenium *taxa, a molecular approach was used in which we analyzed four different chloroplast DNA regions, which were shown to be useful DNA barcodes in past studies [16,18,23,24]. These regions were the *trn*L-UAA intron, *rpo*C, *mat*K and the non-coding spacer region between the two genes *psb*A-*trn*H. Tests were conducted on DNA of three *Parthenium *species (*P. incanum, P. tomentosum*, and *P. schottii*) and three cultivated lines of *P. argentatum *(AZ2, AZ3 and Cal6) (data not shown). The best differentiation of *Parthenium *species and lines within *P. argentatum *was obtained with the *psb*A-*trn*H spacer region barcode. There were 5 indel sites in 400 bp of DNA in the six lines tested. When 1000 bp of the *mat*K DNA barcode were analyzed, a total of 12 indel sites were found. In 600 bp from the *trn*L-UAA intron region, only one indel site was observed. Obtaining good sequence from the *rpo*C spacer region was difficult, but in 500 bp, four indel sites were identified. Therefore, due to the higher number of informative sites, the *mat*K and *psb*A-*trn*H DNA barcodes were used for further studies of *Parthenium *taxa.

### The matK DNA barcode

**Figure 3:**
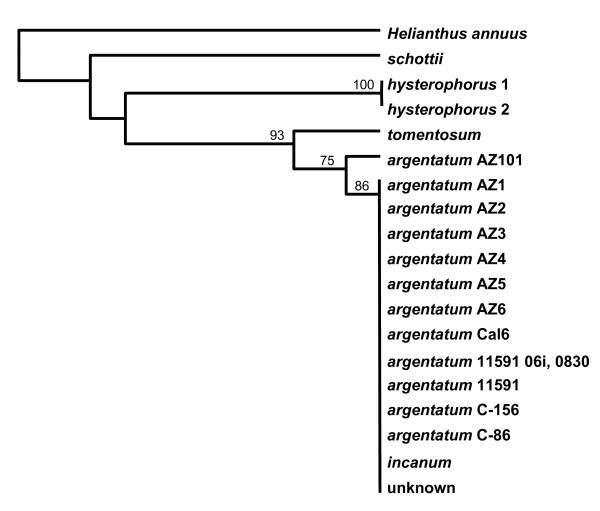
**Differentiation by *mat*K barcode (Genbank Accession **1230803**) in *Parthenium *species**. UPGMA in Jukes-Cantor mode, with gamma correction, was used to construct the tree, with statistical support for tree branches evaluated by bootstrap analysis (1000 replicates), indicated above the node. *Helianthus annuus *is included as an outgroup.

After re-evaluation of the 1000 bp sequence of *mat*K, an efficient barcode for *Parthenium *species was defined. Using the Parth-matK-F and Parth-matK-R primers, *mat*K DNA sequences were examined in *Parthenium *species, lines of *P. argentatum *and AZ101, a hybrid of *P. argentatum *cv. 11591 × *P. tomentosum*. We sampled 601 nucleotides in the *mat*K gene, which yielded fourteen potentially informative, variable positions (2.3%), with eight nucleotide substitutions (1.3%) and six length mutations (indels) (1.0%). Although the *psb*A-*trn*H spacer region in *P. integrifolium *DNA did amplify with the *psb*A-*trn*H barcode primers, the *mat*K locus did not amplify with the *mat*K-barcode primers. This *mat*K barcode was effective at differentiating *P. schottii*, *P. hysterophorus*, and *P. tomentosum *from each other and from a group that included *P. incanum*, *P. argentatum *lines and one hybrid (Figure 3). This barcode did not differentiate *P. incanum *from the seven *P. argentatum *lines and the hybrid (Table 2).

**Table 2:** Population information for analyses of *Parthenium *species using DNA barcode sequences.

			Number of plants tested	
			
*Parthenium *species line/cultivar/hybrid	Seed Harvest year	Location	mat K	psbA-trnH
*argentatum*				
AZ1	2005	MAC^b^	5	21
AZ2	2005	MAC	5	15
AZ2	2006	Higby, AZ	5	16
AZ3	2006	Rush, AZ	5	15
AZ4	2004	MAC	5	15
AZ5	2006	Rush, AZ	5	15
AZ6	2005	MAC	5	20
Cal6	2007	Crit Farm	5	17
C156	2008	MAC	1	1
C86	2008	MAC	1	1
cv. 11591	1989, 2005, 2006	MAC, NALPGRU^c^	13	20
AZ101^a^	2002	USALARC^d^, NALPGRU	3	3
*hysterophorous*	2008	MAC	2	2
*incanum*	2007	USALARC, WRRC	6	6
*integrifolium*	2008	USALARC	-	2
*schotti*	2007	WRRC	1	1
*tomentosum*	2007	USALARC, WRRC^e^	5	5
Unknown	2008	USALARC	1	1

^a^hybrid, *P. argentatum *11591 × *P. tomentosum*

^b^MAC, Maricopa Agricultural Center Field, University of Arizona, Maricopa, AZ

^c^USALARC, US Arid Land Agriculture Research Center Greenhouse, Maricopa, AZ

^d^NALPGRU, National Arid Land Plant Genetic Resources Unit, Parlier, CA

^e^WRRC, Western Regional Research Center Greenhouse, Albany, CA

### The psbA-trnH DNA barcode

**Figure 4:**
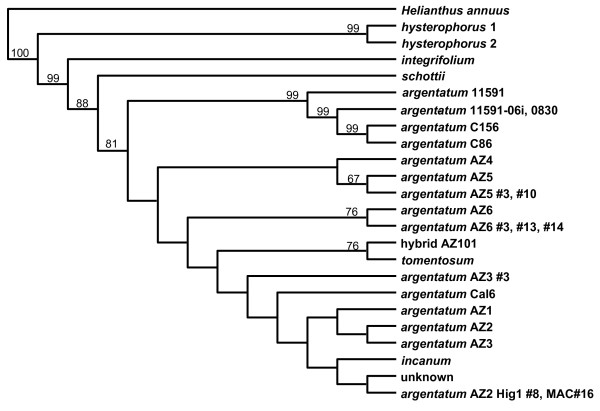
**Differentiation by *psb*A-*trn*H spacer region barcode (Genbank Accession **1230807**)**. This barcode was analyzed in *Parthenium *species, *P. incanum, P. tomentosum, P. schottii, P. integrifolium*, hybrid AZ101 (*P. argentatum *× *P. tomentosum*) and *P. argentatum *lines AZ1, AZ2, AZ3, AZ4, AZ5, AZ6, Cal6, C156, C86 and cv. 11591. UPGMA in Jukes-Cantor mode was used to construct the tree, with statistical support for tree branches evaluated by bootstrap analysis (1000 replicates), indicated above the node. Minority barcodes are indicated by #'s after the name of the line. *Helianthus annuus *is included as an outgroup.

The non-coding spacer region between *psb*A *and trn*H was used to differentiate several *Parthenium *species, lines of *P. argentatum *and a hybrid of two *Parthenium *species (Table 2). A 469 bp region was amplified via PCR using the *psb*A-F and *trn*H-R primers. This region produced the best differentiation (Figure 4). We sampled 456 nucleotides in the *psb*A and *trn*H spacer, which yielded fourteen potentially informative, variable positions (3.1%), with eleven nucleotide substitutions (2.4%) and three length mutations (0.7%). First of all, we found that there was 100% consensus in the barcode sequence among samples tested of line AZ1 (n = 21), AZ4 (n = 15), Cal6 (n = 17), AZ101 (n = 3), *P. incanum *(n = 6) and *P. tomentosum *(n = 5). On the other hand, there was a second barcode sequence within line AZ2 (minority barcode in 6.5% of total, n = 31), AZ3 (minority barcode 6.7%, n = 15), AZ5 (minority barcode 20%, n = 15), AZ6 (minority barcode 15%, n = 20) and 11591 (50% alternative barcode, n = 20). The minority or alternative barcodes differed from the corresponding common barcode by one to three bases.

**Figure 5:**
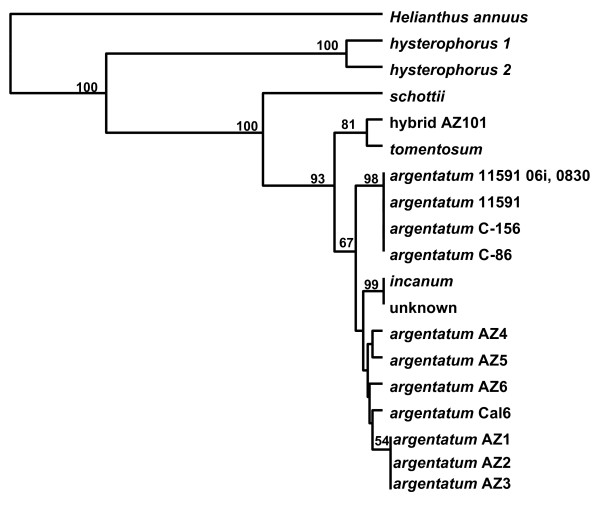
**Barcode differentiation using the combined *mat*K sequence and the spacer region of *psb*A-*trn*H**. Combined barcodes were analyzed in *Parthenium *species, *P. incanum, P. tomentosum, P. schottii*, hybrid AZ101 (*P. argentatum *× *P. tomentosum*) and *P. argentatum *lines AZ1, AZ2, AZ3, AZ4, AZ5, AZ6, Cal6, C156, C86 and cv. 11591. UPGMA in Jukes-Cantor mode was used to construct the tree, with statistical support for tree branches evaluated by bootstrap analysis (1000 replicates), indicated above the node. *Helianthus annuus *was used as an outgroup.

The *psb*A-*trn*H spacer barcode differentiated *P. hysterophorus*, *P. integrifolium *and *P. schottii *from each other and from all the other species and lines. The *psb*A-*trn*H spacer barcode of *P. argentatum *cultivar 11591 and the two breeding lines C156 and C86 was different from those of the remaining *P. argentatum *lines, *P. tomentosum *and *P. incanum*. The barcode of AZ101, which is a hybrid between *P. argentatum *cultivar (cv.) 11591 and *P. tomentosum*, is similar to or identical to that of *P. tomentosum. Parthenium incanum's barcode *clustered with two AZ2 variants and a plant of unknown parentage, indicating their close relationship. Analysis with both the *psb*A-*trn*H spacer and *mat*K barcodes provided further differentiation (Figure 5). The combined barcodes of AZ101 and *P. tomentosum *are more similar to each other than to all those of the *P. argentatum *lines together with *P. incanum*. Drilling deeper, the barcodes of cv. 11591/C156/C86 are different from those of *P. incanum *and all the remaining *P. argentatum *lines.

## Discussion

### Comparative genome organization and structure

Asteraceae is one of the largest families of flowering plants with approximately 1,500 genera and 23,000 species. Production of secondary metabolites is a key feature of this diverse family. For example, several genera within the Asteraceae produce high molecular weight rubber in the cytosol, including *Lactuca sativa *[25] and *Taraxacum kok-saghyz *[26], and the species of interest to our studies, *Parthenium argentatum*. To support efforts to improve the levels of rubber production in this industrial crop, the sequence of the chloroplast genome of *P. argentatum *was determined. This information is useful for our efforts in chloroplast engineering. The barcodes we present will be used in breeding of commercially important lines in the genus *Parthenium*.

Within the Asteraceae, the *P. argentatum *chloroplast sequence represents the fourth complete sequence. This sequence reveals that the chloroplast genomes of *P. argentatum, H. annuus, G. abyssinica *and *L. sativa *are identical in gene order and content (Figure 1; Figure 2). The four genomes differ slightly in length, with the chloroplast genome in *P. argentatum *somewhat longer than those in *L. sativa, G. abyssinica *and *H. annuus*, respectively (Table 1). Two inversions in the chloroplast genome are shared by two of the three subfamilies of the Asteraceae [14,22] and are present in *P. argentatum *(Figure 1). In *H. annuus*, the IR-located gene *ycf*2 has an internal deletion of 455 bp that is not found in the three other genomes. The large chloroplast gene *ycf2 *specifies an expressed protein [27], whose function has not yet been determined, although *ycf*2's homology to ATPases was noted by Wolfe [28]. Our protein domain analysis [29] suggests similarity with conserved domains of the ATPase AAA family that perform chaperone-like functions involved in assembly or disassembly of protein complexes. In some chloroplast genomes, particularly in grasses, *ycf*2 is entirely absent [30]. Despite that fact, knockout studies in *Nicotiana tabacum *demonstrated that *ycf*2 is essential for survival [31]. There must be sufficient coding sequence remaining in *H. annuus *to provide any essential *ycf*2 function. Interestingly, *ycf2 *is one of the eight fastest evolving genes in the chloroplast genome (Additional file 1; [32]). Notably, this rapid evolution has taken place in the framework of the more slowly evolving IR region as a whole (Figure 2; [33]). Another notable size difference in coding regions is found in the SSC region. The SSC region of the chloroplast genome of *P*. *argentatum *is 791 to 1162 bp longer than that in the other species (Table 1). Within the SSC region, the *ycf*1 gene has a 3'-deletion in *H. annuus, G. abyssinica and L. sativa *(Figure 2). Similar to *ycf*2, *ycf*1 encodes a protein of unknown function that is also essential [31]. It appears to be a multi-pass transmembrane protein, with no clear association to known functional domains.

In a comparative study of individual genes of *P. argentatum, H. annuus, G. abyssinica *and *L. sativa*, we identified several sequences with high levels of differences along their length, the most divergent including the already mentioned *ycf*1, and *clp*P, *rps*16, *acc*D, and *ndh*A (Additional file 1). Interestingly, three of these genes, *ycf*1, *acc*D and *clp*P, are essential plastid genes in some taxa, but not others [31,34-37]. The presence of non-coding intronic sequences in both *ndh*A and *rps*16 contributes to the divergence in those two loci [38,39]. These divergent sequences among the four Asteraceae chloroplast genomes identify the fastest evolving regions containing coding sequences.

Metabolic engineering of plants by inserting transgenes in the chloroplast would potentially be made more efficient with knowledge of chloroplast sequences, based on the conclusions of one group that chloroplast transformation efficiency was significantly enhanced when vectors were constructed with 100% homologous sequences [40]. Other groups have shown that precise homology may not be essential, as tobacco sequences [41] were sufficient to allow recombination in tomato [42], potato [43], and petunia [44]. The chloroplast genome sequence of *P. argentatum *was used to design a 100% specific chloroplast transformation vector (unpublished data), to maximize the possibility of successful recombination. Improving crop plants via chloroplast transformation is a viable strategy [1,5] that will be pursued in this industrial crop.

### DNA barcodes

Chloroplast genomic sequences were used to develop DNA barcodes to discriminate at the species level and below. The *mat*K barcode contained sufficient information to differentiate three *Parthenium *species (*tomentosum*, *hysterophorus *and *schottii*) from each other and from *P. argentatum *and *P. incanum*. However, the *mat*K-barcode did not differentiate *P. incanum *from *P. argentatum *or *P. agentatum *lines from each other (Figure 3). The *psb*A-*trn*H spacer barcode provided additional differentiation at the species level and below (Figure 4, 5). Interestingly, when the *ma*tK gene and the *psb*A-*trn*H spacer barcode information was combined, *P. tomentosum *and cv. 11591 were differentiated from the remaining *P. argentatum *lines and *P. incanum*. Using the combined barcodes, we observed that they were more similar in *P. argentatum *AZ1 to AZ6 and Cal6 lines overall than they were in the *P. argentatum *cv. 11591, breeding lines C-156 and C86, and hybrid line AZ101 (Figure 5). To understand the pattern of differentiation, it would be useful to have precise information about the pedigrees of all the lines. Unfortunately, in most cases that is either lacking or incomplete. We know that AZ4 and AZ5 were selected from the same seed lot [45] and their combined barcodes are very similar (Figure 5). We cannot trace the ancestors of AZ4, AZ5 and AZ6 to understand the history of their relatedness to AZ1, AZ2, AZ3 and Cal6. The barcodes of the two *P. argentatum *lines AZ2 and AZ3 were not different, which is not surprising as AZ2 and AZ3 were selections from the same 11591 seed lot [45], however, it would be expected that their majority barcodes would be more similar to 11591 than they are. The *psb*A-*trn*H DNA barcode analysis demonstrated that two plants of AZ2, #8 grown in a field at Higby and #16 grown in a field at the Maricopa Agriculture Center (MAC) have a different *psb*A-*trn*H barcode than the common DNA barcode sequence of AZ2 (Figure 4). These do not appear to be pure AZ2 derivatives and may represent seed contaminants. Several of the *P. argentatum *lines were homogeneous according to the *psb*A-*trn*H spacer sequence, including AZ1, AZ4, and Cal 6. Other lines were less homogeneous, including AZ2, AZ3, AZ5, and AZ6, with a minority sequence present in 6 to 20% of the individuals tested. From our own observations in the field, *P. argentatum *accessions are highly heterogeneous in growth habit, suggesting that seed lots are composed of highly mixed genetic populations. This would not be unexpected for open-pollinated, self-incompatible, field-grown lines. Our barcode data support the heterogeneity and provides information that will be used immediately to differentiate breeding populations.

Classical breeding efforts will be enhanced by using the informative chloroplast DNA barcode we describe herein. We assessed the genetic purity of a small population of *P. argentatum *using the *psb*A-*trn*H barcode and were able to show, as described above, which lines had undergone homogenization and which had not (Figure 5). Knowledge of the purity of lines and the presence of contaminating seeds, will further our breeding efforts of lines that are being advanced for latex production.

Our barcode study was useful in providing support for the maternal parent of the hybrid plant, AZ101. AZ101 is a vigorous interspecific hybrid, low in rubber concentration, but high in biomass production [46]. The line is the result of an open-pollinated cross between *P*. *argentatum *cv. 11591 and *P. tomentosum *cv. stramonium [45]. AZ101 most likely inherited its chloroplast genome from *P. tomentosum*, as AZ101 and *P. tomentosum *are not differentiated by the combined barcode system (Figure 5). Although we do no know the reason for the difference, our results are not the same as those from the non-DNA analyses by Ray and co-workers [47]. More extensive analysis of differences at the DNA level is necessary.

According to the literature, there are about a dozen species of *Parthenium *growing on the North American continent. However, *P. argentatum *is the only species with commercially viable amounts of rubber. Other species such as *P. incanum *and *P. tomentosum *produce primarily resinous materials [48]. The substrate for rubber biosynthesis is isopentenyl pyrophosphate (IPP) [49,50]. Chloroplasts have been shown to contribute to the pool of IPP in plant cells [e.g., [51]; unpublished data, Kumar and Whalen]. If the levels of chloroplastic IPP production vary from line to line, it may be possible to breed for enhancements in substrate production by controlling the maternal parent. This suggests that hybrids could be developed using a maternal parent that produces more rubber like AZ2 combined with a higher biomass from a line like AZ101, to produce a superior plant. More experiments are necessary to understand the role of the maternal parent in rubber biosynthesis.

Our preliminary results on lack of PCR amplification from mature pollen DNA of targets within the IR regions (data not shown), suggest that chloroplasts are not present in the mature pollen and thereby are likely to be maternally inherited in *P. argentatum*. Use of plastid specific barcodes derived from the genome sequence, will allow us to definitively track any paternal inheritance in future experiments. With the recent finding of paternal inheritance in a weedy *Helianthus *species [52], as well as in species previously considered to lack paternal inheritance in pollen, such as *Arabidopsis thaliana *[8,9], it is crucial that extensive studies are performed, especially if a strategy for transgene containment depends on not transferring transgenes in pollen.

## Conclusion

The genome sequence of the *P. argentatum *chloroplast will enrich the sequence resources of plastid genomes in commercial crops. The availability of the complete plastid genome sequence may facilitate improved transformation efficiency by using the precise endogenous flanking sequences and regulatory elements in chloroplast transformation vectors. The DNA barcoding study forms the foundation for genetic identification of commercially important lines of *P*. *argentatum *that are producing natural rubber latex for biomedical applications.

## Methods

### Isolation of chloroplasts and DNA amplification, and sequencing

A mature, greenhouse-grown *Parthenium argentatum *line AZ2 plant was placed in the dark for 2-days before harvesting young leaves. Chloroplasts were isolated from leaves using a 30-52% sucrose-gradient according to both Palmer [53] and Jansen et al. [54]. Genomic DNA from chloroplasts was isolated using the GeneElute Plant Genomic Miniprep kit (Sigma-Aldrich Co.). The resulting DNA was amplified using the REPLI-g whole genome amplification kit (Qiagen, Inc.). Amplified DNA was digested with *Eco*RI and *Bst*BI and examined by agarose gel electrophoresis to confirm the clear banding pattern, which indicated that the amplification product was chloroplast and not nuclear DNA.

### Genome sequencing, assembly and annotation

*Parthenium argentatum *chloroplast genome sequencing was carried out using 454 Sequence Technology (Agencourt Biosciences, Corp). Random sequences were assembled into a draft genome sequence using Newbler as described by Chaisson et al. [55]. The whole genome was annotated using DOGMA (Dual Organellar GenoMe Annotator; [56]) to identify coding sequence, rRNAs, and tRNAs using the plastid/bacterial genetic code. To analyze the similarity of the chloroplast genes in *P. argentatum *and the other members of the Asteraceae, *H. annuus *(NC_007977), *L. sativa *(NC_007578), and *G. abyssnica *(NC_010601), the percent identity of nucleotide sequences within the open reading frame was calculated based on alignments made with ClustalW [57] and BLAST 2 SEQUENCES [58]. Inversions in the chloroplast genome of *P. argentatum *were identified by comparing the sequence in the inversion region [11] with that in *L. sativa, H. annuus *and *Nicotiana tabacum *(NC_001879). The end points of the inversion were determined as described by Timme et al. [22]. The mVISTA program in Shuffle-LAGAN mode [59] was used to compare the DNA sequences of the chloroplast genomes of the four species of Asteraceae, using the sequence annotation information of *P. argentatum *(Figure 2).

### Identification of Parthenium species and lines

To differentiate various *Parthenium *species and lines, a chloroplast DNA barcode system was developed. Four regions of the *Parthenium *chloroplast genome were explored, including the intron in *trn*L-UAA, the *rpo*C and *mat*K genes, and the non-coding spacer between *psb*A-*trn*H. Plant genomic DNA was isolated from young plants (3-4 weeks old) of available *Parthenium *species, cultivars, and lines using DNeasy Plant Mini Kit (Qiagen, Inc.). PCR was carried out with Phusion DNA Polymerase according to manufacturer's instructions (New England Biolabs, Inc.). The primers, TrnL-F, 5'-CGAGTTGGGGATAGAGGGACTTGAAC-3' and TrnL-R, 5'-GATATGGCGAAATAGGTAGACGCTACGGAC-3' were used to amplify *trn*L-UAA; for *rpo*C, rpoC1-F, 5'-CATAGGAGTTGCTAAGAGTCAAATTCGG-3' and rpoC2-R, 5'-CCTTTTCTAGATCTTGATTCACGTAGAAATTCCGC-3'; for *mat*K, matK-F, 5'-GAATTTCAAATGGAGAATTCCAAAGC-3' and matK-end-R, 5'-CGAGCTAAAGTTCTAGCACAAGAAAGTCG-3'; and for *psb*A-*trn*H, psbA-F, 5'-GGAAGTTATGCATGAACGTAATGCTC-3' and trnH-R, 5'-CGCGCATGGTGGATTCACAATC-3'. PCR products were sequenced in both directions. Sequences were compared and any sequences with differences from the majority sequence were re-sequenced in both directions. Barcode differentiations were visualized using the UPMGA best tree method in Jukes-Cantor mode and then bootstrapped with 1000 replicates according to manufacturer's instructions in MacVector (MacVector, Inc.). *Helianthus annuus *was included as an outgroup.

Based on preliminary analysis of selected taxa of *Parthenium*, the central region of the *mat*K gene was the best for finding divergence in *Parthenium *species. DNA from *P. schottii, P. tomentosum*, *P. incanum*, a cultivar of *P. argentatum *cv. 11591, nine lines of *P. argentatum *(AZ1, AZ2, AZ3, AZ4, AZ5, AZ6, C156, C58 and Cal6) and AZ101 (a hybrid of *P. argentatum *cv 11591 × *P. tomentosum*) was amplified via PCR with a 60°C annealing temp, using primers Parth-matK-F, 5'-CAAGCTCATCTGGAAATCTTGGTTCAGGCTC-3' and Parth-matK-R, 5'-GCCAACGATCCAACCAGAGGCATAATTGG-3'. The PCR products were sequenced in both directions using the same primers. In addition, the non-coding spacer region between the two genes *psb*A-*trn*H (500 bp) was used to further differentiate the *Parthenium *taxa. DNA was amplified with the PCR using primers psbA-F and trnH-R at an annealing temperature of 58°C. PCR products were sequenced in both directions with the following primers, psbAF1-seq, 5'-GCTGCTATTGAAGCTCCATC-3' and Rev1-seq-trnh Gua, 5'-CCTTGATCCACTTGGCTACATCCG-3'.

## Abbreviations

IR: inverted repeat; SSC: small single copy; LSC: large single copy; bp: base pair; kb: kilobase pair; INV: inverted region.

## Authors' contributions

SK designed and performed all aspects of the laboratory research, isolated chloroplasts, assembled the genome sequence, compared the coding sequences in the four genomes, designed and performed all barcode amplifications and sequencing, aligned the sequences, and wrote the first draft. FMH conceived of and participated in the sequencing of the chloroplast genome. CMM facilitated all aspects of the laboratory work and revised the manuscript. KC conceived this study, provided the plant lines, and revised the manuscript. MCW supervised the work, assisted in the design of this study, with SK interpreted all data, performed analysis of barcode sequence alignments, and revised all versions of the manuscript. All authors read and approved the final manuscript.

## Supplementary Material

Additional file 1 **Location of *Parthenium argentatum *(Genbank Accession **1230297) **chloroplast genes in the genome sequence**. The coordinates of genes in the chloroplast genome of *Parthenium argentatum *and comparison of the sequence of these genes (% identity) with those in *Helianthus annuus*, *Guitozia abyssinica *and *Lactuca sativa*. Click here for file
