# Supplementary material for: Comparative analysis of the complete sequence of the plastid genome of Parthenium argentatum and identification of DNA barcodes to differentiate Parthenium species and lines
Source: BMC Plant Biol. 2009 Nov 17;9:131. doi: 10.1186/1471-2229-9-131 (PMC2784773; doi:10.1186/1471-2229-9-131)
Supplement: Additional file 1 — Location of Parthenium argentatum (Genbank Accession 1230297) chloroplast genes in the genome sequence. The coordinates of genes in the chloroplast genome of Parthenium argentatum and comparison of the sequence of these genes (% identity) with those in Helianthus annuus, Guitozia abyssinica and Lactuca sativa. [file 1471-2229-9-131-S1.PDF]

Table 1. Location of *Parthenium argentatum* (Genbank Accession 1230297) chloroplast genes in the genome sequence. Comparison with homologues in chloroplast genomes of *Helianthus annuus* (*Ha*), *Guitozia abyssinica* (*Ga*) and *Lactuca sativa* (*Ls*) by percent identity of open reading frames.

				% identity[c]		
**Product Name**	**Locus**	**Start: End[a]**	**Length (bp)[b]**	***Ha***	***Ga***	***Ls***
photosystem II protein D1	psbA	409 : 1467	352	98.9	99.1	98.9
maturase K	matK	2031 : 3542	503	97.4	97.6	94.9
ribosomal protein S16	rps16	4962 : 6069	88	93.3	93.4	92.4
photosystem II protein K	psbK	7445 : 7624	59	97.2	99.4	99.4
photosystem II protein I	psbI	8058 : 8168	36	99.1	99.1	99.1
cytochrome b6/f complex subunit VIII	petN	9287 : 9367	29	100	100	100
photosystem II protein M	psbM	10779 : 10883	34	99.0	97.1	97.1
RNA polymerase beta subunit	rpoB	12901 : 16081	1060	99.2	99.2	95.9
RNA polymerase beta' subunit	rpoC1	16087 : 18913	942	98.5	98.0	96.4
RNA polymerase beta'' subunit	rpoC2	19020 : 23163	1381	97.4	98.3	97.0
ribosomal protein S2	rps2	23452 : 24162	236	99.4	98.9	98.3
ATP synthase CF0 A subunit	atpI	24377 : 25120	247	98.9	99.5	98.3
ATP synthase CF0 C subunit	atpH	26277 : 26522	81	99.6	99.6	99.2
ATP synthase CF0 B subunit	atpF	26899 : 28158	145	98.4	99.1	97.1
ATP synthase CF1 alpha subunit	atpA	28231 : 29757	508	99.1	99.1	97.5
photosystem II protein D2	psbD	32950 : 34009	353	99.4	99.1	98.3
photosystem II 44 kDa protein	psbC	33957 : 35378	473	99.4	99.1	98.3
photosystem II protein Z	psbZ	36069 : 36257	62	99.5	100	100
ribosomal protein S14	rps14	46031 : 46633	100	99.0	100	98.7
photosystem I P700 apoprotein A2	psaB	37510 : 39711	734	99.6	99.2	98.7
photosystem I P700 apoprotein A1	psaA	39740 : 41989	750	98.9	99.4	98.9
photosystem I assembly protein Ycf3	ycf3	42741 : 44708	102	97.3	96.7	95.0
ribosomal protein S4	rps4	46031 : 46633	201	99.3	99.5	97.7
NADH dehydrogenase subunit J	ndhJ	48877 : 49350	150	99.0	99.0	97.9
NADH dehydrogenase subunit K	ndhK	49453 : 50130	225	99.0	98.8	97.9
NADH dehydrogenase subunit 3	ndhC	50184 : 50543	120	98.9	99.4	96.7
ATP synthase CF1 epsilon subunit	atpE	52638 : 53036	133	98.0	98.8	98.0
ATP synthase CF1 beta subunit	atpB	53036 : 54529	499	98.8	98.9	97.5
ribulose-1,5-bisphosphate carboxylase/oxygenase large subunit	rbcL	55303 : 56760	485	98.6	98.4	95.7
acetyl-CoA carboxylase carboxyltransferase beta subunit	accD	57254 : 58762	502	93.8	93.3	90.3
photosystem I subunit VIII	psaI	59516 : 59623	36	99.1	100	99.1
photosystem I assembly protein Ycf4	ycf4	60034 : 60583	184	99.1	98.4	97.1
envelope membrane protein	cemA	61445 : 62131	229	98.6	98.4	97.8
cytochrome f	petA	62371 : 63330	320	98.0	99.0	97.0
photosystem II protein J	psbJ	64203 : 64322	40	100	100	99.2
photosystem II protein L	psbL	64474 : 64587	38	99.9	100	100.0

photosystem II protein VI	psbF	64613 : 64729	39	100	100	99.2
photosystem II protein V	psbE	64742 : 64990	83	100	99.6	99.6
cytochrome b6/f complex subunit VI	petL	66245 : 66337	31	99.0	100	95.8
cytochrome b6/f complex subunit V	petG	66506 : 66616	37	100	99.1	98.2
photosystem I subunit IX	psaJ	67376 : 67501	42	100	98.4	92.6
ribosomal protein L33	rpl33	67955 : 68152	66	98.0	99.0	94.2
ribosomal protein S18	rps18	68332 : 68634	101	99.7	99.3	99.0
ribosomal protein L20	rpl20	68901 : 69281	126	99.2	99.0	97.9
ribosomal protein S12	rps12	70012 : 70125	118	100	100	100
ATP-dependent Clp protease proteolytic subunit	clpP	70306 : 72350	101	91.1	94.1	92.8
photosystem II 47 kDa protein	psbB	72850 : 74370	508	99.4	99.0	97.6
photosystem II protein T	psbT	74582 : 74683	33	98.0	98.0	94.1
photosystem II protein N	psbN	74762 : 74890	42	98.5	98.0	98.5
photosystem II protein H	psbH	74993 : 75211	73	98.2	98.2	96.8
cytochrome b6	petB	75339 : 76765	233	97.4	96.8	95.7
cytochrome b6/f complex subunit IV	petD	76960 : 78167	157	97.7	97.5	97.0
RNA polymerase alpha subunit	rpoA	78368 : 79374	335	98.7	98.4	97.5
ribosomal protein S11	rps11	79481 : 79888	136	98.3	99.0	97.6
ribosomal protein L36	rpl36	79997 : 80107	37	100	100	98.2
Translation initiation factor 1	infA	80225 : 80455	77	99.6	100	98.7
ribosomal protein S8	rps8	80580 : 80981	134	98.0	99.0	97.5
ribosomal protein L14	rpl14	81172 : 81537	122	98.9	98.9	97.6
ribosomal protein L16	rpl16	81650 : 83070	136	99.0	98.0	94.6
ribosomal protein S3	rps3	83229 : 83884	210	98.8	98.0	96.5
ribosomal protein L22	rpl22	83872 : 84333	154	98.1	97.0	93.9
ribosomal protein S19	rps19	83869 : 84333	92	98.1	98.0	97.9
ribosomal protein L2	rpl2	84740 : 86228	183	99.5	97.0	99.5
ribosomal protein L23	rpl23	86247 : 86528	93	100	99.7	99.6
hypothetical chloroplast RF21	ycf2	86879 : 93674	2266	92.3	99.2	97.8
NADH dehydrogenase subunit 2	ndhB	94789 : 96991	510	99.7	99.5	99.6
ribosomal protein S7	rps7	97284 : 97748	155	99.6	99.8	99.1
ribosomal protein S12	rps12	98343 : 98594	118	100	99.0	99.0
hypothetical chloroplast RF1	ycf1	109061 : 113495	1477	83.2	82.3	74.7
ribosomal protein S15	rps15	113864 : 114142	92	97.8	97.1	96.0
NADH dehydrogenase subunit 7	ndhH	114237 : 115415	393	99.1	99.1	98.0
NADH dehydrogenase subunit 1	ndhA	115420 : 117571	338	95.9	96.7	94.2
NADH dehydrogenase subunit I	ndhI	117650 : 118144	166	98.8	98.8	98.0
NADH dehydrogenase subunit 6	ndhG	118515 : 119042	176	99.1	98.7	97.0
NADH dehydrogenase subunit 4L	ndhE	119262 : 119564	101	99.3	99.0	98.4
photosystem I subunit VII	psaC	119806 : 120048	81	99.6	99.6	98.0
NADH dehydrogenase subunit 4	ndhD	120167 : 121666	503	98.4	98.4	97.1
cytochrome c biogenesis protein	ccsA	121911 : 122878	322	98.5	98.6	95.4
ribosomal protein L32	rpl32	123609 : 123773	54	98.2	97.6	95.8
NADH dehydrogenase subunit 5	ndhF	124867 : 127097	743	97.7	97.6	94.7

[a]start-end of open reading frame, with map location based on conventional numbering starting with trnH-GUG at bp 1 and corresponding to that in Figure 1.

[b]length of open reading frame in *P. argentatum*

[c]% identity, the open reading frames of genes were matched manually using Clustal W and NCBI BLAST 2 SEQUENCES programs.
